# Cas1–Cas2 physically and functionally interacts with DnaK to modulate CRISPR Adaptation

**DOI:** 10.1093/nar/gkad473

**Published:** 2023-06-02

**Authors:** Tom Killelea, Juachi U Dimude, Liu He, Alison L Stewart, Fiona E Kemm, Marin Radovčić, Ivana Ivančić-Baće, Christian J Rudolph, Edward L Bolt

**Affiliations:** School of Life Sciences, University of Nottingham, UK; Division of Biosciences, College of Health, Medicine and Life Sciences, Brunel University London, Uxbridge, UK; School of Life Sciences, University of Nottingham, UK; School of Life Sciences, University of Nottingham, UK; School of Life Sciences, University of Nottingham, UK; Department of Biology, Faculty of Science, University of Zagreb, Croatia; Department of Biology, Faculty of Science, University of Zagreb, Croatia; Division of Biosciences, College of Health, Medicine and Life Sciences, Brunel University London, Uxbridge, UK; School of Life Sciences, University of Nottingham, UK

## Abstract

Prokaryotic Cas1–Cas2 protein complexes generate adaptive immunity to mobile genetic elements (MGEs), by capture and integration of MGE DNA in to CRISPR sites. De novo immunity relies on naive adaptation—Cas1–Cas2 targeting of MGE DNA without the aid of pre-existing immunity ‘interference’ complexes—by mechanisms that are not clear. Using *E. coli* we show that the chaperone DnaK inhibits DNA binding and integration by Cas1–Cas2, and inhibits naive adaptation in cells that results from chromosomal self-targeting. Inhibition of naive adaptation was reversed by deleting DnaK from cells, by mutation of the DnaK substrate binding domain, and by expression of an MGE (phage λ) protein. We also imaged fluorescently labelled Cas1 in living cells, observing that Cas1 foci depend on active DNA replication, and are much increased in frequency in cells lacking DnaK. We discuss a model in which DnaK provides a mechanism for restraining naive adaptation from DNA self-targeting, until DnaK is triggered to release Cas1–Cas2 to target MGE DNA.

## Graphical Abstract

**Graphical Abstract:**
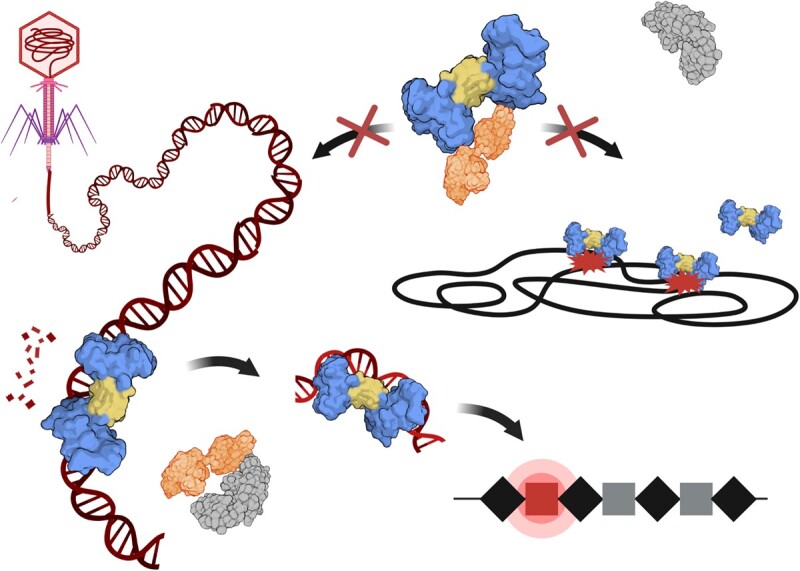

## INTRODUCTION

Prokaryotes utilize specialised chromosomal sites called CRISPRs (Clustered Regularly Interspaced Short Palindromic Repeats) and Cas (CRISPR-associated) proteins to provide adaptive immunity against mobile genetic elements (MGEs). Immunity is generated by CRISPR ‘adaptation’ (1,2), which depends on the Cas1–Cas2 protein complex to capture fragments of MGE DNA (or RNA) and integrate them into a CRISPR as ‘spacers’ (3–6). Cas1–Cas2 captures DNA fragments that are defined by length and end sequences called Protospacer Adjacent Motifs (PAMs) (5–9). (Cas1)_4_–(Cas2)_2_ complexes bind pre-spacer DNA in two Cas1 active sites held either side of the Cas2 dimer, defining the distance between active sites and therefore DNA fragment length. Integration of captured DNA, without its PAM sequence, as a spacer along with synthesis of one new repeat *per* spacer, establishes immunity that is delivered by CRISPR ‘interference’ reactions. Interference depends on transcription of CRISPR to RNA, which is cleaved within the repeat regions into crRNAs representing a single spacer, and which are bound into interference complexes (Cascade–Cas3 in *E. coli*) (10). These survey DNA for PAMs, ‘locking’ into an R-loop where a PAM is at MGE sequence complementary to the crRNA, triggering nuclease destruction of the MGE (11–14). PAMs therefore provide functional coupling of adaptation with interference, for effective immune responses.

Interaction of Cas1–Cas2 with interference nucleases temporally and spatially targets adaptation to MGE DNA, providing new DNA fragments for capture (15–17). This is ‘primed’ adaptation (17,18), which therefore relies on pre-existing CRISPR immunity that has already generated spacer-crRNAs. But if there is no pre-existing immunity ‘naive’ adaptation by Cas1–Cas2 generates immunity de novo, by targeting MGE DNA independently from interference nucleases by mechanisms that are unclear. When Cas1–Cas2 is over-expressed in cells ectopically (e.g. from an inducible plasmid) in the absence of interference complexes it readily derives new spacers from the host chromosome. This is in accord with the PAM preference of Cas1–Cas2 (ATG in *E. coli*) for sequences abundant across host chromosomes and MGEs, but does not provide for targeting of MGE DNA (19). Host proteins that assist DNA capture by Cas1–Cas2, including RecBCD helicase (20–22), and RecJ, DnaQ and Cas4 nucleases (23–25), do not appear to contribute to Cas1–Cas2 distinguishing MGE DNA as a target (20,26). We report multiple lines of evidence indicating that in *E. coli* the widely conserved ‘hub’ chaperone DnaK (Hsp70) (27,28) regulates naive adaptation by restraining Cas1–Cas2. This protects the host chromosome from targeting by Cas1–Cas2. We show that inhibition of adaptation can be reversed by mutation of DnaK and by expression of MGE protein. This may provide DNA target selection to MGEs, when Cas1–Cas2 is released from DnaK that is recruited by MGE proteins.

## MATERIALS AND METHODS

### Strains, plasmids and media

*Escherichia coli* strains are described in the Supplementary Table S7. We generated an *E. coli* Δ*dnaK* strain by recombineering (29) to insert kanamycin resistance followed by P1 *vir* transduction into BW25113 (30). The Δ*dnaK* phenotype was confirmed *via* plaque formation and temperature sensitivity tests. Cells were grown at 37°C in LB broth (10 g/l bacto-tryptone, 5 g/l yeast extract, 10 g/l NaCl) and on LB agar plates (supplemented with 15 g of agar/L for solid media) unless otherwise stated. Antibiotics were added to LB plates at final concentrations: tetracycline 10 μg/ml, ampicillin at 100 μg/ml, and chloramphenicol at 34 μg/ml. Plasmids are detailed in Supplementary Table S8. Briefly, pBad-HisA (Invitrogen) was used for expression of Cas1–Cas2 under control of arabinose inducible araBAD promoter as in previous studies (30).The plasmid pACYCduet (Novagen) was used for expression of DnaK and other proteins, each under control of the IPTG inducible T7 promoter.

### BioID2 identification of Cas1-Cas2 interactor proteins

*E. coli* strain EB377 (Table S7) was transformed with pCas1^BioID2^–Cas2 or pBioID2 and grown on ampicillin agar. Individual colonies were used to inoculate LB medium supplemented with ampicillin and 0.2 (w/v) % L-arabinose and grown for 18 h to provide starter cultures. These starter cultures were used to inoculate LB supplemented with 0.2% (w/v) L-arabinose. Cells were grown for 60 min prior to harvesting at 4000 × *g* for 5 min. Biomass was washed three times with 10 ml of 1 × PBS and then resuspended in 1 ml of Lysis Buffer (50 mM Tris pH 7.5, 150 mM NaCl, 0.4% SDS, 1% Nonidet P40, 1.5 mM MgCl_2_) for lysis by sonication and clarification by centrifugation at 16 000 × *g* for 15 min. Lysates were incubated with Pierce™ High-Capacity Streptavidin Agarose Beads (Thermo Scientific™) overnight at 4°C with gentle agitation. Beads were washed three times in 1 × PBS to remove unbound protein, and sent to the Cambridge Centre for Proteomics for analysis using a 120-min LC–MS/MS run, with the resulting raw data available accompanying this work (Table S1). The NSAF value for each protein was determined as the number of spectral counts (SpC) identifying a protein, divided by the protein's length (L), divided by the sum of SpC/L for all protein in the experiment (31).

### *In-vivo* co-expression and pull down of a DnaK-Cas1 complex

Strain EB377 was co-transformed with p^His^DnaK and pCas1^Strep^–Cas2, or respective empty vector controls, and grown on agar with antibiotic selection. Overnight cultures containing antibiotics were inoculated with single colonies and grown for 18 h to provide starter cultures for growth to OD_600_ of 0.6 prior when cells were treated with 0.2% (w/v) L-arabinose and IPTG to 1 mM to induce Cas1, Cas2 and DnaK protein expression. After 3 h cells were harvested and resuspended in 1 ml of Pull-Down Buffer (20 mM Mops pH7, 200 mM NaCl) supplemented with phenylmethylsulfonyl fluoride (PMSF) (0.1 μM final concentration). Cells were lysed by sonication and clarified by centrifugation at 16 000 × *g* for 30 min, and lysate incubated for 60 min at 4°C with gentle agitation following addition of 50 μl of Iminodiacetic acid Sepharose^®^ (Merck) pre-charged with NiCl_2_. Samples washed 3 × 1 ml of Pull-Down Buffer with 50 mM imidazole, were heat treated in 1 x SDS Denaturation Buffer (50 mM Tris pH 6.8, 2% SDS, 10% Glycerol, 0.1 M DTT, 6 M urea, 0.5 M imidazole, bromophenol blue). Sample separation used a 12.5% SDS gel and transferred onto Amersham™ Hybond™ P 0.2 PVDF membrane (Cytivia™). Standard western blotting methods were followed with membrane blocked in Blocking Buffer (3% milk powder in 1 × TBS–Tween). Primary antibodies: Mouse 6×-His Tag Monoclonal Antibody (HIS.H8), Biotin (Invitrogen™) and Mouse Anti-Strep-tag II mAb Monoclonal Antibody (MBL^®^), and secondary antibodies: Goat Anti-Biotin HRP-linked, and Goat anti-Mouse IgG (H + L) Secondary Antibody HRP, all added at 1:2000 dilution in blocking buffer. The membrane was treated with ECL Western Blotting Substrate (Promega™) and imaged using a LAS-3000 mini (FUJIFILM™).

### Naive adaptation assays and corresponding measurement of plasmid instability and cell viability

Naive adaptation assays were based on the procedure described in (1). *E. coli* EB377 cells transformed with plasmid vector lacking Cas1–Cas2 (pControlA), pEB628 (pCas1–Cas2) or pTK145 (pCas1R84G–Cas2) were inoculated into 5 ml of LB and aerated at 37°C for 16 h in LB containing 0.2% (w/v) L-arabinose, and then sub-cultured (‘passaged’) by diluting 1:300 into fresh LB again supplemented with 0.2% (w/v) l-arabinose. Cells were harvested at identical time points and genomic DNA extracted using a GeneJET Genomic DNA Purification Kit (Thermo Scientific™). Spacer acquisition was monitored by PCR, utilizing 10 ng of genomic DNA and primers SW1 and SW2 (Supplementary Table S9), with products separated using a 1.25% agarose gel stained with ethidium bromide and imaged using a U:Genius3 (Syngen Biotech). For naive adaptation assays expressing Cas1–Cas2 alongside other proteins, strain EB377 was co-transformed with pEB628 and pACYC plasmids containing the gene of interest. Cells were passaged as described above and stopped in P2 at OD_600_ 0.4, with spacer acquisition monitored by PCR as described above. Analysis of acquisition was carried out from three independent replicates, with band quantification of PCRs carried out with ImageJ (32). Plasmid instability during naive adaptation assays was analysed by comparison of cell viability on LB and antibiotic selection plates. At the end of each ‘passage’ samples were taken and serially diluted in 1 × M9 Minimal Salts. 10 μl of each dilution was spotted onto LB-agar plates with and without selection and grown overnight prior to colony quantification.

### Protein purification

Cas1 was purified with a C-terminal StrepTag^®^II (Cas1^Strep^), Cas2 with an N-terminal StrepTag^®^II (^Strep^Cas2), and DnaK proteins with an N-terminal hexahistidine tag (^His^DnaK). Individual transformants of BL21-AI cells containing the relevant plasmid were used to prepare fresh overnight cultures which were subsequently diluted 1:100 in 3 l of LB supplemented with selection marker. Cells were grown to an OD_600_ of 0.6 and protein expression induced by addition of IPTG (1 mM) and L-arabinose (0.02% w/v). After 3 h cells were harvested and resuspended in Buffer A (20 mM Tris pH 7.5, 150 mM NaCl and 10% glycerol) supplemented with PMSF to 0.5 mM. Cas1^Strep^ and ^Strep^Cas2 were individually loaded onto 5 ml Strep-Avidin™ XT Superflow™ High-Capacity Cartridges (IBA Life Sciences GmbH) in Buffer A before being eluted via an isocratic elution in Buffer A supplemented with 50 mM biotin. Cas1^Strep^ was further purified using a 1 ml HiTrap Heparin Hp (Cytivia™) in Buffer A and eluted in a gradient of 0.15–1 M NaCl. Cas1^Strep^ containing fractions were loaded onto a HiLoad 16/600 Superdex 200 pg (Cytivia™) equilibrated in Buffer A. Cas1^C-Strep^ was concentrated using a VivaSpin^®^ 6 10 kDa cut-off centrifugal concentrator (Sartorius) prior to storage at –80°C. ^Strep^Cas2 was further purified by loaded onto a 1 ml HiTrap Q XL column (Cytivia™) and collected in the flow through before being dialysed overnight at 4°C against 20 mM Tris pH 7.5, 150 mM NaCl and 25% glycerol prior to storage at –80°C.

^His^DnaK and mutant variants were loaded onto a 5 ml HiTrap Chelating HP (Cytivia™) and washed with Buffer B (20 mM Tris pH 7.5, 500 mM NaCl, 20 mM imidazole, 10 mM MgCl_2_ and 5 mM ATP and 10% glycerol), before elution in Buffer A using a gradient of 20–500 mM imidazole. ^His^DnaK containing fractions were dialysed overnight at 4°C against Buffer C (50 mM Tris pH 7.5, 100 mM NaCl, 1 mM DTT and 10% glycerol) before loading a 5 ml HiTrap Q HP (Cytivia™) in Buffer C and eluted with a gradient of 100–1000 mM NaCl with ^His^DnaK containing fractions dialysed overnight at 4°C in 50 mM Tris pH 7.5, 50 mM NaCl, 1 mM DTT and 20% glycerol prior to storage at –80°C.

### *In-vitro* spacer integration assays

Spacer integration (SpIn) assays were carried out in a reaction buffer containing 20 mM HEPES–NaOH pH 7.5, 25 mM KCl, 10 mM MgCl_2_, 1 mM DTT and 0.1 mg/ml BSA. Reactions were prepared to a final volume of 10 μl, with the relevant concentrations of proteins used in each assays indicated in the relevant figure or figure legend. Cas1^Strep^, or Cas1^Strep^ and ^Strep^Cas2, were left to incubate on ice for 10 min in reaction buffer prior to addition of ^His^DnaK, followed by an additional 5 min incubation on ice. Reactions were initiated by addition of 20 nM Cy5-labelled Pre-spacer (formed from annealed TK24 and TK25, Supplementary Table S10) and 100 ng of supercoiled pCRISPR (Supplementary Table S8). Reactions were immediately transferred to 37°C for 60 min, before being quenched by addition 1 μl of Stop Buffer (0.2 mg/ml proteinase K, 2% SDS and 100 mM EDTA), and left for a further 60 min at 37°C. 1× DNA loading dye (2.5% Ficol 400, 3 mM Tris pH 8.0, 10 mM EDTA, 0.08% SDS, Orange G) was added to samples prior to loading onto a 1.5% agarose TAE gel and left to migrate for 60 min at 120 V before imaging on a Typhoon™ laser scanner platform (Cytivia™). All gel images were processed using ImageJ.

### Electrophoretic mobility shift assays

Cas1 and DnaK proteins were diluted to working concentrations in 20 mM Tris pH 8.0, 100 mM NaCl and 1 mM DTT prior to addition. Cas1 was preincubated for 5 min with 20 nM Cy5 labelled DNA fork substrate and 20 mM Tris pH 8.0, 0.1 mg/ml BSA, 7% Glycerol. DnaK was added to the reaction followed by glycerol to 25% and left for a further 25 min at 37°C. Samples were loaded onto a 5% native acrylamide gel and left to migrate for 90 min before imaging on a Typhoon™ laser scanner platform. All gel images were processed using ImageJ.

### Single-image microscopy

Fresh overnight cultures of strains of interest were diluted 100-fold in fresh LB broth supplemented with ampicillin (50 μg/ml) if required and incubated with vigorous aeration at 37°C until *A*_600_ reached 0.2. l-Arabinose (Sigma) was added to a final concentration of 0.1% and the culture incubated 60 min for protein expression and maturation. If necessary, the DNA dye Hoechst 33342 (Invitrogen™) was added to a final concentration of 200 ng/ml, incubated for 5 min at room temperature and imaged without washes. 1 μl of the culture was pipetted onto an agarose pad and air-dried. For generation of pads a 65 μl (15 × 16 mm) GeneFrame (Thermo Scientific™) was added to a conventional microscopy slide. 1% of SeaKem LE agarose (Lonza) was added to 1 × M9 minimal medium (diluted from a 5 × stock, Sigma-Aldrich) and heated until the agarose was completely dissolved. 95 μl of the solution was added into the GeneFrame chamber and the chamber sealed immediately with a conventional microscopy slide. Once set, the top slide was removed and the agarose pad air-dried for no more than 5 min at 37°C and used immediately. Once the sample was added and air-dried the GeneFrame chamber was sealed by adding a 22 × 22 mm cover slip. Visualisation was by using a T*i*-U inverted microscope (Nikon) with a CFI Plan Fluor DLL 100 × objective (Nikon) and an ORCA Flash 4.0 LT plus camera (Hamamatsu). Phase contrast images were taken using a pE-100 single LED wavelength source (CoolLED). For fluorescence the pE-4000 illumination system (CoolLED) was used. The relevant filters for visualisation of DAPI, eYFP and mCherry were Nikon DAPI-50LP-A, Zeiss filter set 46 (eYFP), as well as Nikon TXRED-A-Basic Filter (mCherry). Images were captured using the NIS Elements-BR software V4.51 (Nikon) and exported to tiff. Postprocessing, such as cropping and rotating, was performed in Adobe Photoshop CC (V23.0.0).

### Time-lapse microscopy

Fresh overnight cultures of strains of interest were diluted 100-fold in fresh M9 with 0.4% (v/v) glycerol, supplemented with ampicillin (50 μg/ml) if required. M9 minimal medium was used for time-lapse experiments to avoid the auto-fluorescence typical for LB broth, which requires longer exposure times and consequently much more rapid photo-bleaching of the fluorophores. Glycerol was used as carbon source because glucose would repress the arabinose-controlled promoter. Cultures were incubated with vigorous aeration at 37°C until OD_600_ reached 0.2. l-Arabinose (Sigma) was added to a final concentration of 0.1% and the culture incubated 60 min for protein expression and maturation. 1 μl of the sample was pipetted onto an agarose pad and air-dried. Pads were generated as described above. Once the sample was added and air-dried the GeneFrame chamber was sealed by adding a 22 × 22 mm cover slip. Cells were visualised using the T*i*-U system described above. The temperature was maintained at 37°C using an environmental chamber (Digital Pixel). Time-lapse stacks were captured using the NIS Elements-BR software V4.51 (Nikon) and either exported to a single mp4 file or individual tiff files. Postprocessing of tiff images, such as cropping and rotating, was performed in Adobe Photoshop CC (V23.0.0).

## RESULTS

### DnaK physically interacts with Cas1 and inhibits naive adaptation by Cas1-Cas2 in *E. Coli*

**Figure 1.**
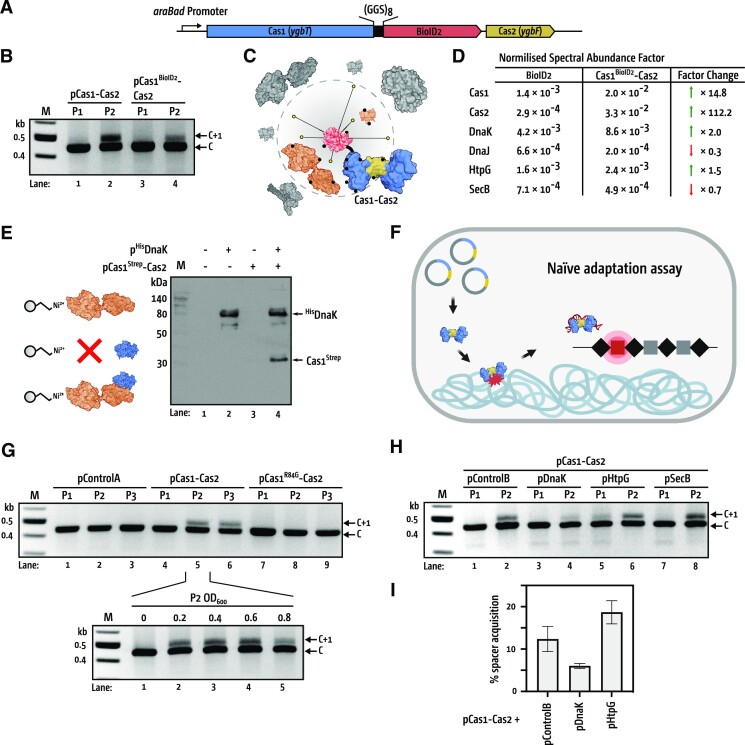
Physical and functional interaction of Cas1–Cas2 with DnaK. (**A**) The pCas1^BioID^–Cas2 expression construct fusing Cas1 at its C-terminus to biotin-protein ligase *via* a (GGS)_8_ tripeptide linker. (**B**) Cas1^BioID^–Cas2 supports naive adaptation in *E. coli* (lanes 3 and 4), compared with Cas1–Cas2 control (lanes 1 and 2). Genomic DNA was extracted from cells during the 1st and 2nd passages (P1 and P2) and naive adaption detected from expanded chromosomal CRISPR-1 (C + 1) that has acquired at least one new spacer (32 bp) and a repeat (29 bp). (**C**) Proteomics by the proximity-dependent labelling method; BioID2 (red) fused to Cas1 in Cas1–Cas2 complex, blue, and yellow respectively, activates biotin (yellow dots) which conjugates (black dots) to proteins in the labelling radius of BioID2. Biotinylated proteins (orange) are enriched from cells by streptavidin binding, separating them from unlabeled proteins (grey). (**D**) Normalized Spectral Abundance Factor values for Cas1, Cas2 and chaperone proteins identified by peptide fingerprinting in biotinylated protein samples enriched from cells expressing BioID2 (control) or Cas1^BioID2^–Cas2 during growth in 50 μM biotin media. (**E**) Western blot detection of ^His^DnaK and Cas1^Strep^ after affinity purification pull down following *in-vivo* co-expression of Cas1^strep^–Cas2 complex and ^His^DnaK. (**F**) Summary of naive acquisition assays. Multicopy plasmid inducibly expresses Cas1–Cas2 in *E. coli* cells, providing Cas1 (blue)–Cas2 (yellow) complex that captures DNA from the chromosome, and integrate it into the *E. coli* CRISPR-1 locus (C + 1). (**G**) Representative agarose gel for PCR-based detection of CRISPR-1 expansion (C + 1) across P1-P3, by Cas1–Cas2 complex, compared with an empty vector control (pControlA) and inactive Cas1^R84G^–Cas2 complex. The mean (n = 3) percentage of spacer acquisition is shown at the bottom of the gel—Supplementary Table S2 shows raw data. The lower panel shows monitoring of CRISPR-1 expansion (C + 1) by Cas1–Cas2 at the specific P2 OD readings indicated (0–0.8). (**H**) Representative agarose gel summarizing PCR-based detection of CRISPR-1 expansion (C + 1) during co-expression of Cas1–Cas2 alongside *E. coli* chaperones. pCas1–Cas2 was co-transformed into cells with either the pControlB (pACYCDuet empty vector) or pACYCDuet containing one of *dnaK* (pDnaK), *htpG* (pHtpG) or *secB* (pSecB). (**I**) Quantification of naive adaptation in cells expressing DnaK and HtpG during P2. Supplementary Table S4 shows the raw data measurements of spacer acquisition.

We investigated for interactors of Cas1–Cas2 in replicating *E. coli* cells by using BioID2, a proximity-dependent biotin protein labelling technique (33) that we adapted for *E. coli*. Cas1 was fused at its C-terminus *via* a tri-peptide repeat (GGS)_8_ linker to the biotin-protein ligase R40G mutant from *Aquifex aeolicus*, under inducible control and alongside Cas2 in the same plasmid (Figure 1A). The resulting protein complex (Cas1^BioID2^–Cas2) catalysed naive adaptation in *E. coli* cells (Figure 1B). Proteins entering physical proximity to induced Cas1^BioID2^–Cas2 were biotinylated in cells growing in 50 nM biotin (summarised in Figure 1c), providing a biotinylated proteome differing from control cells expressing only the biotin-protein ligase (Supplementary Figure S1a). Streptavidin extraction of the biotinylated proteins from these proteomes, followed by peptide mass fingerprinting and normalised spectral abundance factor (NSAF) analysis of these proteins (31), identified enriched DnaK in the Cas1^BioID2^–Cas2 sample (Figure 1D and Supplementary Table S1), compared with the control. This suggested physical interaction of DnaK with Cas1^BioID2^–Cas2, consistent with a previous *E. coli* proteomics study that identified physical interaction of DnaK with YgbT protein, now called Cas1 (28). We further validated this physical interaction by co-expressing ^His^DnaK and Cas1^Strep^–Cas2 from plasmids in *E. coli* cells, alongside controls expressing strep-tagged or (His)_6_-tagged other than Cas1 and DnaK (Figure 1e and Supplementary Figure S1b). Trapping ^His^DnaK on Ni-NTA resin also extracted Cas1^Strep^ in a stable complex, but not Cas2 or a strep-tagged control protein (^Strep^POLD2), confirmed by SDS-PAGE and western blotting (Figure 1E and Supplementary Figure S1b).

To assess whether DnaK modulated naive adaptation in *E. coli*—which depends on Cas1–Cas2—we measured acquisition of new DNA spacers into the chromosomal CRISPR-1 locus. Cas1–Cas2 was inducibly over-expressed from a plasmid, to overcome repression of chromosomal Cas1 (*ygbT*) by H-NS (1,6,21,30,34). In naive adaptation assays Cas1–Cas2 captures DNA from the chromosome (21,22) and integrates it as spacers, observed as expansion of the chromosomal CRISPR-1 site, summarised in Figure 1f. Naive adaptation was detectable in passages two and three of *E. coli* cell growth (P2 and P3) observed across optical densities compared with control cells containing plasmid lacking Cas1 (*ygbT*) and Cas2 (*ygbF*) (Figure 1G, lanes 5 and 6). Cells expressing Cas1^R84G^–Cas2 complex, in which Cas1 cannot bind to DNA (30), gave no detectable naive adaption (lanes 7–9), confirming naive adaptation dependent specifically on plasmid Cas1–Cas2. Cell viabilities were similar in P2 for each *E. coli* cell type grown in ampicillin or not (Table S3), confirming that plasmid instability or cell death is not responsible for observed differences in spacer acquisition (Supplementary Figure S1c and d). Co-expressing DnaK alongside Cas1–Cas2 inhibited naive adaptation in P2, compared with cells expressing Cas1–Cas2 alongside the empty plasmid expression vector for *dnaK* (Figure 1H and I). HtpG, a chaperone that modulates Cas3 in *E. coli* (35), and SecB, which has no connection with CRISPR systems, did not inhibit naive adaptation (Figure 1H and I). We conclude that DnaK and Cas1 physically interact in *E. coli* cells, and that DnaK inhibits naive adaptation by Cas1–Cas2.

### Naive adaptation is released from inhibition by mutation or deletion of DnaK, and by expression of MGE proteins

**Figure 2.**
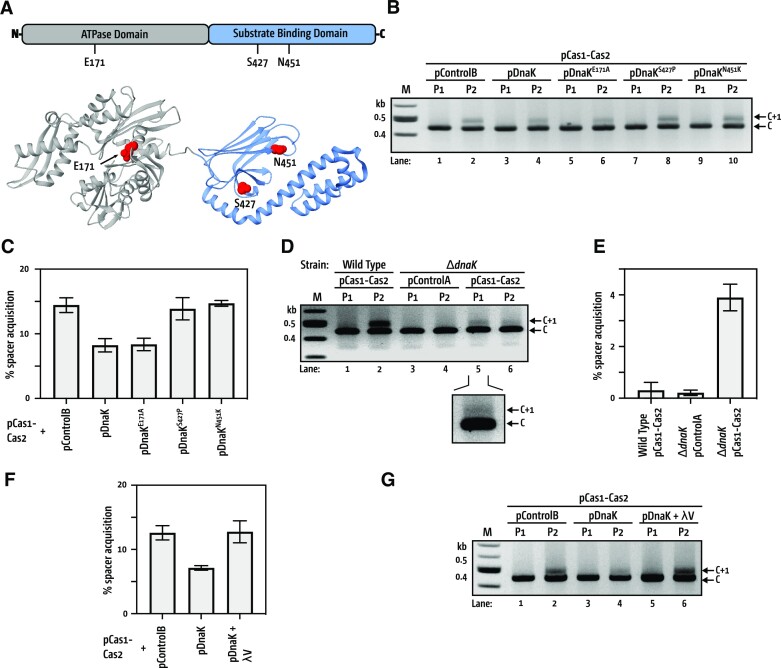
DnaK inhibits naive acquisition. (**A**) Summary of DnaK functional domains (from PBD: 2KHO (50)). The ATPase domain (grey) and Substrate Binding domain (blue) show in red the positions of E171 required for ATP hydrolysis, and S427 and N451 that are in the peptide binding cleft and are required for DnaK to inhibit naive adaptation. (**B**) Representative agarose gel summarizing PCR-based detection of CRISPR-1 expansion by Cas1–Cas2 in P1 and P2 when co-expressed alongside wild type DnaK or mutants as indicated. (**C**) Plots of the mean (*n* = 3) percentage of spacer integration, and Supplementary Table S5 presents the raw data. (**D**) Representative agarose gel summarizing PCR-based detection of CRISPR-1 expansion by Cas1–Cas2 expressed in wild type and Δ*dnaK* cells as indicated at top of the gel. (**E**) Measurement of Cas1–Cas2-dependent new spacer acquisition during P1 in wild type and Δ*dnaK* cells—raw data is in Supplementary Table S6. Standard error bars are shown for mean values from *n* = 3. (**F**) Measurement of Cas1–Cas2-dependent new spacer acquisition during P2 in cells expressing DnaK alone or DnaK alongside proteins λP or λV as indicated. Values are means with standard errors from *n* = 3, with (**G**) a representative gel for the data shown.

DnaK binds to and releases proteins from its C-terminal Substrate Binding Domain (SBD), triggered by allosteric modulation of its N-terminal ATP hydrolysis domains, summarised in Figure 2A, reviewed in (27). By introducing mutations that deactivate either the SBD (DnaK^S427P^ or DnaK^N451K^) or ATPase (DnaK^E171A^) sites (36) and co-expressing the mutant proteins with Cas1–Cas2 we investigated how DnaK inhibits naive adaptation (Figure 2b and c). DnaK^E171A^ was as effective as DnaK^+^, but DnaK^S427P^ or DnaK^N451K^ released naive adaptation from inhibition (Figure 2B and C). Viability of cells expressing each mutant were similar (Supplementary Figure S1e) and western blotting confirmed that each DnaK mutant protein was detectably expressed similarly to DnaK^+^ during the assays (Supplementary Figure S1e).

Deletion of chromosomal *dnaK* (Δ*dnaK*) in cells expressing Cas1–Cas2 was therefore predicted to also de-repress naive adaptation. *E. coli dnaK* was deleted from the chromosome by recombineering (29) and the resulting cells were phenotyped at the commencement of each experiment to avoid suppressor mutations that compensate for Δ*dnaK*—Δ*dnaK* cells should be unable to support phage λ infection, and show temperature sensitivity (Supplementary Figure S2a–c). Expression of Cas1–Cas2 in wild type cells gave naive adaptation in P2, as expected (Figure 2d lanes 1 and 2), but Cas1–Cas2 in Δ*dnaK* cells gave additional naive adaptation within P1, compared with no naive adaptation in Δ*dnaK* cells lacking Cas1–Cas2 expression (Figure 2D, lanes 3–6, and Figure 2E), and despite Δ*dnaK* cell populations showing 10,000-fold lower cell viability than wild type cells (Supplementary Figure S2d). Measurement of naïve adaptation from each cell population in P1 (Figure 2D, lane 5 and Figure 2E) was from identical amounts of extracted genomic DNA (10 ng), detailed in methods, providing controlled comparison of adaptation in each cell type despite differences in cell viabilities. The compromised viability of Δ*dnaK* cells and their overall sickness may explain the much-reduced naive adaptation in P2, compared with the wild type cells, but we conclude that mutations or loss of DnaK stimulated naive adaptation by Cas1–Cas2 to occur in P1.

We next tested for physiological conditions that release naive adaptation from inhibition. DnaK undergoes dynamic binding and release cycles when chaperoning ‘client’ proteins, including when DnaK is ‘hijacked’ by MGE proteins for DNA replication and quality control for assembly of phage particles (27,28,37). Specific interactions of proteins from phage λ with DnaK in *E. coli* are essential for the λ lytic cycle—exemplified in figure S2a—therefore we co-expressed λ proteins from the same plasmid as DnaK, and alongside Cas1–Cas2, and assessed naive adaptation. Expression of λV protein restored fully functioning naive adaption compared with control cells expressing DnaK alone alongside Cas1–Cas2 (Figure 2F, G and Supplementary Table S7). λV protein forms phage coat/tail structures, and has not previously been shown to interact with DnaK. We also tested λP protein, which is required to interact with DnaK for initiating λ phage replication (38–40), and observed modest de-repression of naive adaptation that was not as effective as λV protein (Supplementary Figure S2e and f, and Supplementary Table S7).

### Cas1 foci require DNA binding and are controlled by DnaK

**Figure 3.**
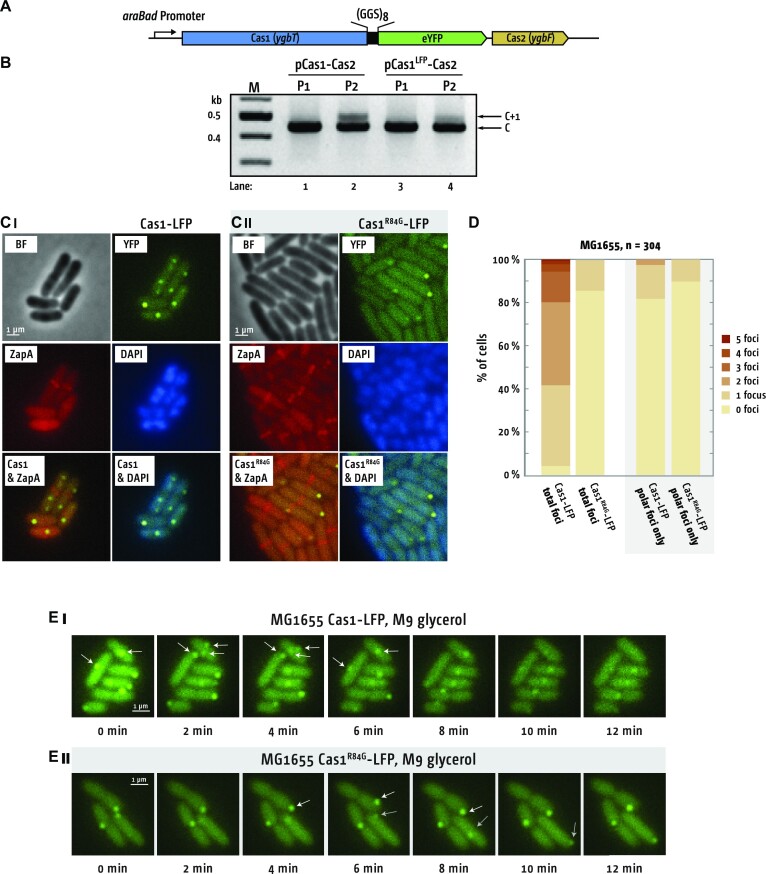
Cas1 foci dynamics in *E. coli* cells. (**A**) Illustration of Cas1-LFP + Cas2 construct that generated functional Cas1–Cas2 complex for imaging in cells. (**B**) The Cas1-(GSS)_8_ linker-eYFP (Cas1-LFP) expression construct catalyzes naive adaptation in *E. coli* cells (lanes 3 and 4), shown compared with Cas1–Cas2 control (lanes 1 and 2) for P1 and P2. The gel shows naive adaption detected as expanded chromosomal CRISPR-1 (C + 1). (**C**) Localization of (i) Cas1-LFP–Cas2 and, (ii) the Cas1^R84G^ -LFP–Cas2 mutant in cells. Sites of future cell division are highlighted by a ZapA-mCherry fusion protein, which allows visualization of the Z-ring. The nucleoid is visualized by staining the cells with Hoechst 33342 (‘DAPI’). (**D**) Foci counts in cells expressing Cas1-LFP and Cas1^R84G^-LFP. The left pair of stacked columns shows direct comparison of total foci from imaging Cas1-LFP and from Cas1^R84G^-LFP, as indicated. The right pair of stacked columns shows only cells that exclusively showed foci at the cell poles. Data were obtained from two independent experiments from a total of 304 cells for Cas1 and Cas1^R84G^. (**E**) Time lapse microscopy of foci from (i) Cas1-LFP and (ii) Cas1^R84G^-LFP, each co-expressed with Cas2. To avoid the auto-fluorescence of LB to minimize photo-bleaching, cells were grown in M9 medium with 0.4% glycerol to early exponential phase. An environmental chamber was used to maintain a constant temperature of 37°C and cells imaged for 15 min, with frames taken every 2 min. The movement patterns of some foci is highlighted by white and grey arrows and see also Supplementary Movies A & B).

To observe the behaviour of Cas1–Cas2 in living cells, and whether it is influenced by DnaK, we fused Cas1 to the fluorophore eYFP. By fusing Cas1 at its C-terminus to eYFP *via* a (GGS)_8_ linker (Cas1-LFP, Linker Fluorescent Protein), and with Cas2 inserted downstream of eYFP for co-expression from the same plasmid (Cas1-LFP + Cas2, Figure 3A)—therefore similarly to the Cas1-BioID2 fusion—we were able to detect naive adaptation in cells, confirming that Cas1-LFP + Cas2 was functioning (Figure 3B). Cas1-LFP produced bright foci corresponding with the space occupied by the nucleoid (Figure 3C, panel I). Replacing Cas1 in the Cas1-LFP + Cas2 construct with Cas1^R84G^, which does not bind stably to DNA (30), altered the frequencies and localisation of foci (Figure 3c panel II and Figure 3D). Only 15% of Cas1^R84G^-LFP expressing cells showed foci, compared with 96% of cells expressing Cas1, which also often (>50% of cells) showed multiple foci (Figure 3C and D). Most Cas1^R84G^-LFP foci (70%) were located in the cell poles, rather than in nucleoid stained areas, compared with 19% of Cas1-LFP foci (Figure 3C, panel II). Imaging the foci in real-time revealed that the non-polar Cas1-LFP foci are mobile between distant locations, but the majority of Cas1^R84G^-LFP polar foci were generally much reduced in mobility, (Figure 3E and Supplementary Movies A and B). Our imaging data is consistent with Cas1-LFP focus formation that is strongly promoted by Cas1 binding to DNA.

**Figure 4.**
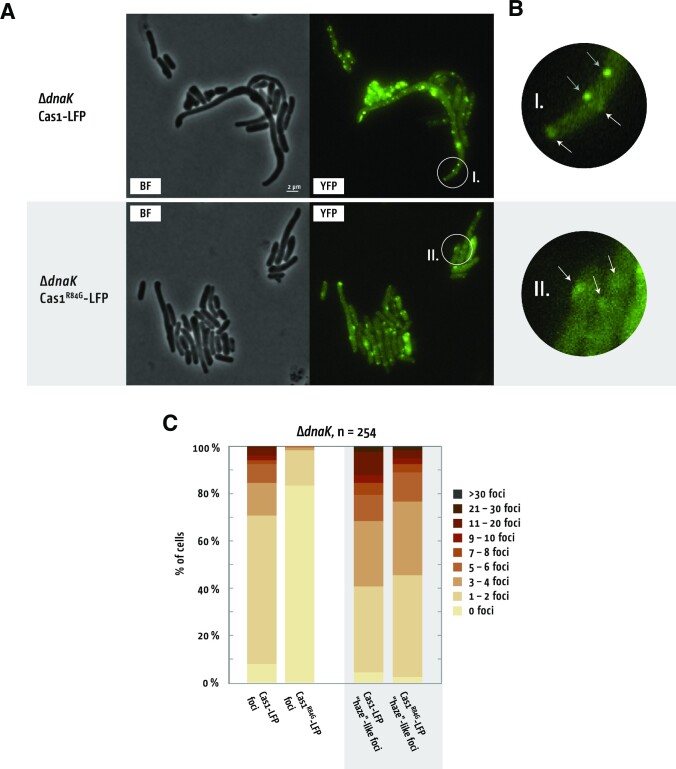
Cas1 foci behavior alters in the absence of DnaK. (**A**) Δ*dnaK* cells were grown to early exponential and expression of Cas1-LFP or Cas1^R84G^-LFP (along with Cas2) was induced for 60 min before visualization. (**B**) Magnifications of the images in panel A, with exact locations in panel A highlighted by white circles labelled I. and II. White arrows show large foci that might be aggregated protein, while grey arrows highlight foci that have a similar appearance than the foci in wild type cells (see also Figure 3A). (**C**) Foci counts in living Δ*dnaK* cells expressing Cas1-LFP and Cas1^R84G^-LFP. The left pair of stacked columns shows direct comparison of counts classed as foci, the right pair of columns shows direct comparison of larger and less regularly shaped foci, called ‘haze’-like. Data were obtained from 2 independent experiments from a total of 254 cells for Cas1-LFP and 254 cells for Cas1^R84G^-LFP.

Cas1-LFP foci in Δ*dnaK* cells were dramatically changed in frequency and distribution compared with wild type cells (Figure 4), with no foci observed in Δ*dnaK* cells expressing only eYFP (Supplementary Figure S3). Cells with multiple foci increased to 90%, with 8% of Δ*dnaK* cells generating greater than 10 foci (Figure 4C). Increased frequencies of well-defined Cas1-LFP foci in Δ*dnaK* cells was accompanied by additional ‘classes’ of foci, highlighted encircled (I) in Figure 4A and B. The additional foci formed a larger area and showed uneven distribution of brightness, which we describe as ‘haze-like’ (Figure 4B, highlighted). When haze-like foci were included in focus counting > 60% of cells had multiple foci (Figure 4C). When we assessed Cas1^R84G^-LFP foci in Δ*dnaK* cells we observed substantially reduced in frequency; 85% of cells showed no foci at all, compared with < 10% of Δ*dnaK* cells expressing ‘wild-type’ Cas1-LFP (Figure 4C). When present, the Cas1^R84G^-LFP foci present were consistently always haze-like in morphology, localised throughout the cell (Figure 4A and B). Thus, normal-looking foci were specifically reduced if Cas1^R84G^-LFP was expressed, while only minor changes were observed for the haze-like foci (Figure 4C). The behaviour of Cas1-LFP foci compared with foci from Cas1^R84G^-LFP, and the stimulatory effects of Δ*dnaK* on naive adaptation and focus formation, are consistent with DnaK restraining Cas1–Cas2 from DNA capture.

### Cas1 foci form during active DNA replication

**Figure 5.**
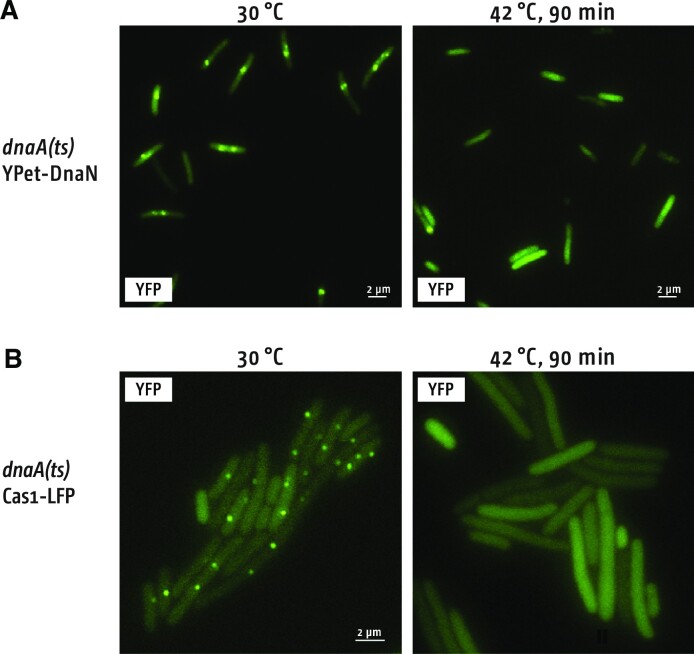
Formation of Cas1-LFP foci requires activated DNA replication. (**A**) *dnaA46* cells express a temperature sensitive DnaA replication initiator protein that is unable to initiate DNA replication forks at *oriC* at 42°C. Temperature control of replisome formation is visualized using the replisome sliding clamp protein (DnaN) fused to the fluorophore YPet, which form foci representing each bi-directional replication fork at 30°C but no foci at 42°C. (**B**) *dnaA46* cells were grown at 30°C to early exponential phase. The culture was then split, and half incubated at 30°C before imaging, with the induction of Cas1-LFP expression (Cas2 being co-expressed) for 60 min before imaging. The other half was shifted to 42°C before imaging, with the induction of Cas1-LFP expression for 60 min.

Naive adaptation in *E. coli* has been suggested to target active DNA replication forks (21). To investigate this using Cas1-LFP foci we generated cells conditionally unable to support DNA replication, but which can be switched over to active DNA replication, predicting that this may alter the presence and absence of foci. *E. coli* cells were synchronised using a temperature-sensitive allele of the replication initiation protein DnaA (*dnaA46*) that can initiate replication at *oriC* at 30°C, but cannot at 42°C. Therefore shifting cells from 30°C to 42°C prevents new DNA replication, while all ongoing rounds of replication reach termination. A fluorescent version of the DnaN replisome sliding clamp protein (YPet-DnaN), was used to monitor the number of replisomes in cells. At 30°C cells showed robust DnaN foci corresponding to the replisome, which were absent after a 90 min incubation period at 42°C (Figure 5A), confirming this as effective for preventing new DNA replication initiating. We then visualised Cas1-LFP by splitting an exponentially growing culture. One half was incubated at 30°C, while the other half was shifted to 42°C. After each culture had been incubated for 30 min, arabinose was added to induce Cas1-LFP + Cas2 expression. Both cultures were then incubated for 60 further min at their respective temperatures before cell imaging. Cells that remained at 30°C showed robust numbers of Cas1-LFP foci, but no foci were observed in cells shifted to 42°C (Figure 5B). This provides direct support for Cas1 targeting DNA during active DNA replication.

### DnaK inhibits DNA binding and integration by Cas1 *in vitro*

**Figure 6.**
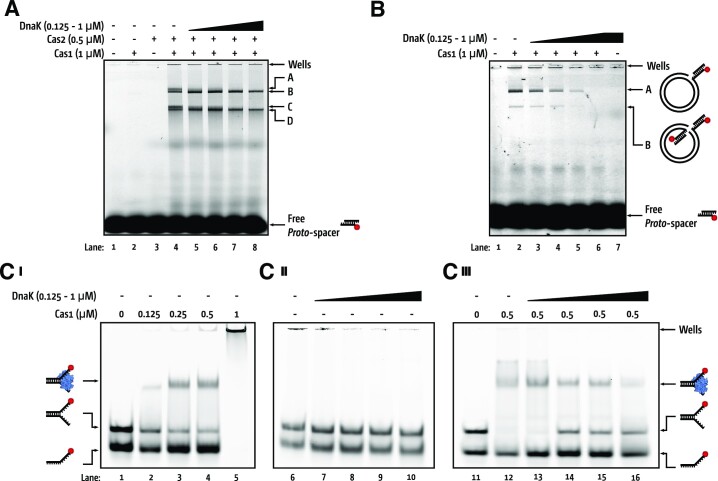
Purified DnaK protein inhibits DNA binding and catalysis by Cas1 and Cas1–Cas2. (**A**) Cas1–Cas2 catalyzed DNA integration was inhibited by increasing concentrations of DnaK. Cas1 and Cas2 at concentrations indicated were preincubated for 10 min prior to addition of DnaK (0–1 μM), Cy5-labelled *proto*-spacer and unlabeled supercoiled pCRISPR. Reactions were for 60 min at 37°C and products analyzed by migration on a 1.5% agarose gel. Free Cy5-DNA, and Cy5-labelled integration products A, B, C, and D are indicated. (**B**) Cas1 *proto*-spacer half-site integration was inhibited by increasing concentrations of DnaK. Cas1 was pre-incubated with increasing concentrations of DnaK prior to addition of Cy5 labelled DNA and unlabeled supercoiled pCRISPR. Reactions were left for 60 min at 37°C and analyzed by migration on a 1.5% agarose gel. Free Cy5-Pre-spacer, and Cy5-labelled integration products A and B are indicated to the right of gel. **(C)** Panels i–iii show EMSAs of increasing concentrations Cas1, DnaK or Cas1 and DnaK as indicated incubated with Cy5-ssDNA and a Cy-5 labelled flayed DNA duplex. In panel III Cas1 was pre-incubated with Cy5 labelled fork before addition of DnaK. Cy5 labeled single stranded DNA, fork, and fork–Cas1 complex are indicated to the sides of panels. All gels were 5% acrylamide, imaged using a Typhoon™ laser scanner platform (Cytivia).

We investigated the mechanism for DnaK inhibiting naive adaptation by physical interaction targeting Cas1 (Figure 1E). Naive adaptation in *E. coli* requires that the Cas1–Cas2 complex binds to a DNA fragment and integrates it as a new CRISPR spacer (8). On formation of Cas1–Cas2 complex from individually purified Cas1 and Cas2 proteins (Supplementary Figure S4a) we observed integration of a model ‘protospacer’ Cy-5 labelled DNA fragment (Suppl. Table 11) into a supercoiled plasmid—formation of DNA products required both Cas1 and Cas2 (Figure 5A, compare lanes 2 and 3 with lane 4). Titration of DnaK inhibited Cas1–Cas2 (Figure 6A, lanes 5–8) to the extent that no product A or C were formed when DnaK was equimolar to Cas1 (1 μM, lane 8). DnaK also fully inhibited Cas1 alone, consistent with pull-down data (Figure 1E and Supplementary Figure S2b), in reactions detecting Cas1 catalysed integration of the same model Cy-5 labelled protospacer into a plasmid to form either nicked or linearised DNA plasmid visible from the integrated Cy5 moiety (41) (Figure 6B).

We then assessed whether DnaK influenced Cas1 DNA binding to flayed duplex DNA in complexes sufficiently stable to observe in EMSAs (22,42). Providing Cas1 with a mixture of Cy-5 end labelled ‘decoy’ ssDNA and flayed duplex substrate of the same sequence, formed a stable complex with only the flayed duplex DNA (Figure 6c i lanes 2–4) with Cas1 eventually aggregating DNA into the gel wells (i, lane 5). DnaK did not bind to DNA in EMSAs, as expected, (Figure 6Cii, lanes 6–10), but DnaK titration (0.25–1 μM) into pre-bound Cas1 (0.5 μM)–DNA complexes, released flayed duplex DNA from binding by Cas1, including DNA from gel wells (Figure 6Ciii, lanes 11–16). When we replaced DnaK with purified DnaK^S427P^ (Supplementary Figure S4b), DnaK with the SBD mutation that was ineffective at inhibiting naïve adaptation, we observed Cas1 prebound to DNA was not removed, consistent with this DnaK mutant being unable to associate with Cas1 (Supplementary Figure S4c and d). These *in vitro* data are consistent with DnaK being able to restrain naive adaptation by preventing Cas1-DNA binding, and provides a plausible explanation for the altered behaviour of Cas1-LFP foci in cells.

## DISCUSSION

Our work identifies that chaperoning of Cas1–Cas2 by DnaK in *E. coli* cells regulates naive adaptation. The influence of DnaK on multiple biological processes across prokaryotes is reflected in its widespread conservation, and we now reveal that DnaK physically and functionally interacts with and controls a CRISPR system. We detected it using BioID-based proteomics, which was validated by physical interaction between DnaK and Cas1 that was sufficiently robust to extract Cas1 from Cas1–Cas2 complex when they are expressed together in cells (Figure 1). This revealed the inhibitory effect of DnaK on naive adaptation only when we inducibly expressed DnaK from a plasmid, to ‘compete’ with Cas1–Cas2 that was also inducibly expressed from a plasmid—in typical naive adaptation assays any effect of DnaK is not noticed, likely because levels of plasmid-induced Cas1–Cas2 overwhelm the levels of chromosomally encoded DnaK, resulting in self DNA targeting by Cas1–Cas2 that is typical of naive adaptation assays. The robust Cas1-DnaK interaction was also apparent from DnaK removing Cas1 from DNA to which it was pre-bound *in vitro*. Prokaryotic CRISPR immunity systems all deploy Cas1–Cas2 to generate immunity, and DnaK is highly conserved across all prokaryotes, except in hyperthermophilic archaea, and across diverse CRISPR types—DnaK residues Ser-427 and Asn-451 that we observed are required for inhibition of naive adaptation are conserved in DnaK in prokaryotes with all CRISPR systems types, I-VI. We suggest that DnaK may control CRISPR adaptation beyond the Type IE *E. coli* CRISPR system.

**Figure 7.**
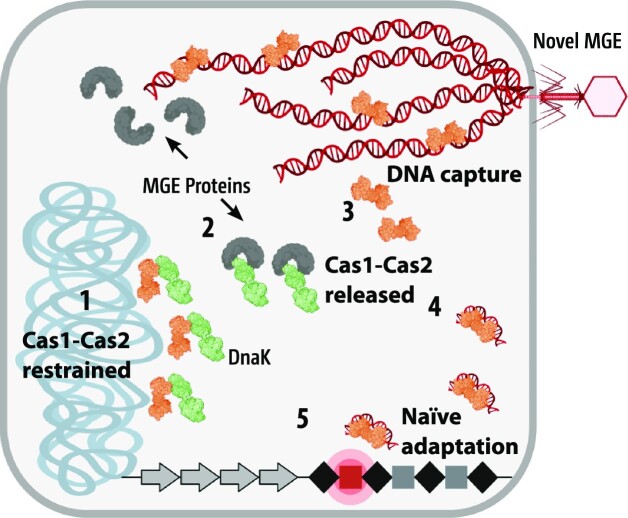
A model for naive adaptation restrained and released by Cas1–Cas2. **1 –** DnaK restrains Cas1–Cas2 and naive adaptation through physical interaction with the DnaK substrate binding domain, protecting the host chromosome until, **2 –** invader MGE expresses proteins that recruit DnaK to assemble MGE DNA replication. **3 –** This releases Cas1–Cas2 from DnaK in allosterically controlled DnaK protein binding and release cycles, **4 –** bringing Cas1–Cas2 into physical proximity with sites of replicating MGE, where DNA capture leads to **5 –** integration as new spacers in CRISPR sites.

Inhibition of naive adaptation by DnaK raises the question of how Cas1–Cas2 may be released for DNA capture, and if this may provide targeting of Cas1–Cas2 to MGEs. We observed that inhibition of naive adaptation by DnaK was reversed in the presence of phage λ proteins. Recruitment of DnaK to plasmid and phage MGE proteins is required to initiate their DNA replication and to control quality and chaperoning of phage head and tail packaging proteins (27,43–49)—the essential role of DnaK is evident from the inability of phage λ to infect *E. coli* Δ*dnaK* cells (Supplementary Figure S2a). These processes are controlled by dynamic binding and release of DnaK client proteins from the substrate binding domain, governed by ATP-hydrolysis triggered allosterically by other proteins, including the DnaJ co-chaperone, recently reviewed (27). Physical interaction of DnaK and Cas1 may be a binding and release process to protect the chromosome from targeting by Cas1–Cas2, until an MGE client protein of DnaK triggers release of Cas1–Cas2. This may spatially and temporally control Cas1–Cas2 to target MGE DNA that is generated in abundance during invader MGE genome replication. In this model (Figure 7), Cas1–Cas2 would not be *distinguishing* MGE DNA from host chromosomal DNA, but is instead restrained from self-targeting and released to target MGEs through interaction with DnaK. Our observation that Cas1 foci can be triggered by active DNA replication, but are not detected when we suppress initiation of replication (Figure 5), raises an intriguing possible mechanism for Cas1–Cas2 targeting to MGE DNA, when MGE proteins suppress host chromosomal replication in preference for triggering that of the MGE genome. Our data from several lines of investigation provide new insights about how DNA is targeted so that CRISPR immunity can be established. It newly reveals interplay between natural CRISPR systems and a host factor (DnaK) that is a central controller ‘hub’ for other cellular processes, including of DNA replication. Further investigation will be able to identify whether the DnaK–Cas1 interactions that we have identified are common across CRISPR systems, and similarly control adaptation.

## Supplementary Material

gkad473_Supplemental_Files

## Data Availability

The mass spectrometry proteomics data have been deposited to the ProteomeXchange Consortium via the PRIDE partner repository with the dataset identifier PXD042090.
