# Supplementary material for: Cas1–Cas2 physically and functionally interacts with DnaK to modulate CRISPR Adaptation
Source: Nucleic Acids Res. 2023 Jun 2;51(13):6914–26. doi: 10.1093/nar/gkad473 (PMC10359635; doi:10.1093/nar/gkad473)
Supplement: gkad473_Supplemental_Files [file gkad473_supplemental_files.zip › Suppl Material - Text-Tables-Figures.pdf]

Supplementary Figure Legends

Figure S1:

A) Western blot comparing biotinylated protein profiles from expressing BioID2 Cas1^{BioID2}-Cas2 or BioID2 control in cells grown in LB media supplemented with 50 µM biotin for 60 minutes. Anti-biotin antibody was used to detect the presence of biotinylated proteins. M = Biotinylated protein marker.

B) SDS-PAGE showing that pull-down of ^{His}DnaK by Ni-NTA resin is accompanied by physical interaction with Cas1^{Strep} (lane 10, and see also main Figure 1e), but a 'mock' pull-down replacing Cas1^{strep} with another strep-tagged protein (^{Strep}Control, ^{Strep}POLD2 [7]) resulted in no interaction with ^{His}DnaK (lane 8). In addition, neither Cas1^{Strep} nor ^{Strep}POLD2 controls expressions bound non-specifically to the Ni-NTA resin, when expressed alone, without ^{His}DnaK (lanes 7 and 9). Lanes 1-5 show total protein profiles from the over-expression cells, confirming the presence of each protein in biomass mixed with Ni-NTA resin.

C) Histogram showing the average number (n=3) of viable cells/ml with standard error of the mean indicated, for the duration of a 3-passage (P1–P3) naïve acquisition assay.

D) Cell viability 'spot tests' during standard naïve spacer acquisition assay conditions. Representative samples are shown, taken at the end of each passage of cell growth, on LB or Ap (ampicillin) agar.

E) Histograms showing viability of *E. coli* cells expressing the DnaK proteins as indicated growing in either LB or LB supplemented with ampicillin

F) Confirmation that the DnaK mutant proteins were expressed from plasmids during naïve acquisition. Biomass samples were taken at 16h (P1) and 4 h (P2) and analyzed via western blot using anti His Tag Antibody-Biotin, and Anti Biotin, HRP-linked antibody as the primary and secondary detection antibodies. The location of DnaK is indicated to the right of the gel with a black arrow. M = Biotinylated protein marker.

Figure S2: Phenotype validation of *E. coli* ∆*dnaK*

A) λ phage plaque assay on lawns of either MG1655 or ∆*dnaK*. Clear zones of lysis are observed for λvir grown on MG1655 but as expected for cells lacking DnaK, the λ phage cannot replicate and therefore plaques are not formed. The λ dilution factor is shown for each panel.

B) Assessment of temperature sensitivity of *ΔdnaK* cells, compared to M1655 following growth for 18 h at either 37 °C or 42 °C.

C) Complementation test assessing the temperature sensitivity of *ΔdnaK* cells when expressing DnaK from a plasmid following growth for 18 h at either 37 °C or 42 °C.

D) Histogram for mean number (n=3) of viable cells/ml with standard error of the mean indicated for wild type (EB377) and *ΔdnaK* (EB410), during 2 passages of a naïve spacer acquisition assay.

E) Representative agarose gel summarizing PCR-based detection of CRISPR-1 expansion by Cas1-Cas2 in P1 and P2 when co-expressed with DnaK alone or DnaK alongside proteins λP or DnaB as indicated.

F) Measurement of Cas1-Cas2-dependent new spacer acquisition during P2 in cells expressing DnaK alone or DnaK alongside proteins λP or DnaB as indicated. Values are means with standard errors from n = 3, except for pDnaK + DnaB (n=1), raw data is in Supplementary Table 7.

Figure S3. eYFP expression in *ΔdnaK*

Cells were grown in LB (Miller) to early exponential phase. eYFP was induced for 60 min before visualization by adding arabinose.

Figure S4. Purified proteins used for *in-vitro* assays

A) Purified Cas1^{Strep} and Cas2^{Strep} migrated on a 15 % SDS gel and visualized by Coomassie staining.

B) Purified ^{His}DnaK and ^{His}DnaK^{S427P} proteins visualized by Coomassie staining in a 12.5 % SDS gel.

C) See also Figure 2a; summary of the DnaK protein domain organization highlighting the positioning of the Ser-427 residue that was changed to proline in this work.

D) See also Figure 6c; lanes 1-6 show an EMSA using forked DNA (lane 1) bound by Cas1 (0.5 μM, lanes 2-6) that is not displaced by ^{His}DnaK^{S427P} added to reactions at the concentrations shown, after binding of Cas1 to DNA. Lanes 7-11 are controls confirming that ^{His}DnaK^{S427P}, like wild type DnaK, does not bind to DNA in these assays.

Supplementary Movies

Movie A – *E. coli* MG1655 cells expressing Cas1-LFP and Cas2 show mobile foci measured over time (see details in main text), compared with **Movie B** showing *E. coli* MG1655 cells expressing Cas1^{R84G}-LFP, showing foci that are immobilized to cell poles.

SUPPLEMENTARY TABLES

Supplementary Table 1: BioID2 mass spec raw data is presented in the accompanying Excel file.

Supplementary Table 2: Naïve acquisition assay band quantification for control data. Expanded CRISPR locus was calculated as a percentage of total PCR product from repeat sample (n=3), with the standard error of the mean shown in brackets for each sample.

		% Expansion of CRISPR locus		
E. coli Strain	Plasmid	P1	P2	P3
	pControlA	0	0	0
EB377	pCas1-Cas2	0	21.3 (1.3)	21.2 (3.4)
	pdCas1-Cas2	0	0	0

Supplementary Table 3: Cell viability during acquisition assays. Mean number of spots (n=3) with standard error of the mean (SEM) included, for serially diluted cells upon the end of an acquisition passage when plated on LB-agar and Ampicillin-LB-agar.

LB-Agar

E. coli Strain	Plasmid	P1 Cells/ml	P1 (SEM)	P2 Cells/ml	P2 (SEM)	P3 Cells/ml	P3 (SEM)
EB377	pControlA	5.8E+09	4.5E+08	5.3E+10	2.0E+09	3.1E+09	4.3E+08
	pCas1-Cas2	2.2E+09	1.2E+08	9.0E+10	8.5E+09	2.3E+09	2.8E+08
	pdCas1-Cas2	1.2E+09	1.0E+08	4.8E+10	3.6E+09	6.2E+09	2.6E+08

Ampicillin-Agar

E. coli Strain	Plasmid	P1 Cells/ml	P1 (SEM)	P2 Cells/ml	P2 (SEM)	P3 Cells/ml	P3 (SEM)
EB377	pControlA	6.9E+09	3.6E+08	4.5E+10	3.0E+09	1.9E+09	3.5E+08
	pCas1-Cas2	2.4E+09	2.6E+08	8.6E+10	9.1E+09	5.2E+08	8.2E+07
	pdCas1-Cas2	1.6E+09	3.3E+07	1.1E+10	8.8E+08	3.9E+08	3.5E+07

Supplementary Table 4: Naïve acquisition assay band quantification values for pCas1-Cas2 when expressed with DnaK or alternative chaperone proteins. Expanded CRISPR locus was calculated as a percentage of total PCR product from repeat sample (n=3), with the standard error of the mean shown in brackets for each sample.

	Plasmids	% Expansion of CRISPR locus	
E. coli Strain	pCas1-Cas2 +	P1	P2
EB377	pControlB	0	12.3 (3.0)
	pDnaK	0	6.0 (0.6)
	pHtpG	6.0 (1.0)	18.7 (2.7)
	pSecB	0	20.7 (2.3)

Supplementary Table 5: Naïve acquisition assay band quantification values for pCas1-Cas2 when expressed with DnaK or DnaK functional mutants. Expanded CRISPR locus was calculated as a percentage of total PCR product from repeat sample (n=3), with the standard error of the mean shown in brackets for each sample.

	Plasmids	% Expansion of CRISPR locus	
E. coli Strain	pCas1-Cas2 +	P1	P2
EB377	pControlB	0.0	14.4 (1.1)
	pDnaK	0.0	8.2 (1.0)
	pDnaK^{E171A}	0.0	8.4 (1.0)
	pDnaK^{S426P}	0.0	13.9 (1.7)
	pDnaK^{N451K}	0.0	14.7 (0.5)

Supplementary Table 6: Naïve acquisition assay band quantification values for pCas1-Cas2 when expressed in EB377 and *ΔdnaK*. Expanded CRISPR locus was calculated as a percentage of total PCR product from repeat sample (n=3), with the standard error of the mean shown in brackets for each sample.

		% Expansion of the CRSIPR locus	
E. coli Strain	Plasmids	P1	P2
EB377	pCas1Cas2	0.3 (0.3)	16.4 (3.4)
Δ*dnaK*	pControlA	0	0
	pCas1Cas2	3.9 (0.5)	1.2 (0.8)

Supplementary Table 7: Naïve acquisition assay band quantification values for pCas1-Cas2 when expressed with DnaK, or DnaK and phage λ P / V protein. Expanded CRISPR locus was calculated as a percentage of total PCR product from repeat sample (n=3), with the standard error of the mean shown in brackets for each sample, unless indicated by * in which case n=1.

	Plasmids	% Expansion of the CRSIPR locus	
E. coli Strain	pCas1-Cas2 +	P1	P2
EB377	pControlB	0	12.6 (1.1)
	pDnaK	0	7.1 (0.4)
	pDnaK + λ P	0	8.9 (1.2)
	pDnaK + λ V	0	12.8 (1.7)
	pDnaK + DnaB	0	9.1*

Supplementary Table 8: *Escherichia coli* K-12 strains

Strain number	Relevant Genotype[a]	Source/Construction/Reference
**General P1 donors**		
BL21AI™	*araB::T7RNAP-tetA*	Invitrogen™
MR37	BW25113 *ΔdnaK::<kan>*	This study[b]
RRL190	AB1157 *<kan>-ypet-dnaN*	[1]
RRL232	AB1157 *zapA-mCherry<cat>*	David Sherratt
**MG1655 derivatives**		
MG1655	F^{-} *rph-1*	[1]
AS1057	*zapA-mCherry<cat>*	MG1655 × P1.RRL232 to Cm^{r}
AS1062	*<kan>-ypet-dnaN*	MG1655 × P1.RRL190 to Km^{r}
AU1054	*dnaA46 tnaA::*Tn*10*	[2]
EB377	*araB::T7RNAP-tetA*	MG1655 × P1.BL21AI to Tc^{r}
EB410	*ΔdnaK::<kan>*	EB377 × P1.TK098 to Km^{r}
JD1460	*dnaA46 tnaA::*Tn*10 <kan>-ypet-dnaN*	This study[c]
JD1685	*ΔdnaK::kan* pMR6	MR37 × pMR6 to Ap^{r}
JD1708	MG1655 pTK135	MG1655 × pTK135 to Ap^{r}
JD1716	MG1655 pTK136	MG1655 × pTK136 to Ap^{r}
JD1722	*ΔdnaJ::<cat>*	This study[d]
JD1725	*ΔdnaJ::<cat>*	MG1655 × P1.JD1722 to Cm^{r}
JD1759	*zapA-mCherry<cat>* pTK135	AS1057 × pTK135 to Ap^{r}
JD1760	*zapA-mCherry<cat>* pTK136	AS1057 × pTK136 to Ap^{r}
JD1765	*ΔdnaJ::<cat>* pTK135	JD1725 × pTK135 to Ap^{r}
JD1766	*ΔdnaJ::<cat>* pTK136	JD1725 × pTK136 to Ap^{r}
JD1776	*dnaA46 tnaA::*Tn*10* pTK135	AU1054 × pTK135 to Ap^{r}
JD1781	*ΔdnaK::kan*	MG1655 × P1.1685 to Km^{r}
JD1783	*ΔdnaK::kan* pTK135	JD1781 × pTK135 to Ap^{r}

JD1784	*ΔdnaK::kan* pTK136	JD1781 × pTK136 to Ap^{r}
RCe690	MG1655 pECR25	MG1655 × pECR25 to Ap^{r}
TK098	*ΔdnaK::kan* pMR6	MR37 × pMR6 to Ap^{r}

a – Only the relevant additional genotype of the derivatives is shown. The abbreviations *kan* and refer to insertions conferring resistance to kanamycin (Km^{r}) and chloramphenicol (Cm^{r}). Tn*10* indicates the presence of a transposon 10 integration, which confers resistance to tetracycline (Tc^{r}). ‘<>’ indicates the use of *frt* sites, where *frt* stands for the 34 bp recognition site of the FLP/*frt* site-directed recombination system. Thus, *<kan>* refers to a kanamycin marker flanked by an *frt* site either side.

b – Constructs were generated by using the one-step method for the inactivation of chromosomal genes, as described [3]. The kanamycin resistance cassette was amplified from pKD4 using primers dnaKdel F/R. To make P1*vir* lysate, Δ*dnaK* cells were transformed with the pMR6 plasmid and then infected by P1*vir* phage.

c – Constructs were generated by using the one-step method for the inactivation of chromosomal genes, as described [3]. For the construction of JD1460, purified genomic DNA from AS1062 was used to amplify the deletion construct using primers 5’dnaN_for and 3’dnaN_rev. A conventional P1*vir* transduction is not possible, as *dnaA* and *dnaN* are genetically linked.

d - Constructs were generated by using the one-step method for the inactivation of chromosomal genes, as described [3], with the kanamycin resistance cassette amplified from pDIM141 [4] using primers dnaJ-catdel-fw and dnaJ-catdel-rv.

Supplementary Table 9: Plasmids used in this study

Plasmid	Name	Description/Reference/Commercial Manufacturer
pControlA	pBadHisA	Invitrogen™
pControlB	pACYCDuet	Novagen®
pETDuet		Novagen®
peYFP	pLau18	eYFP containing plasmid, AddGene
pCas1-Cas2	pEB628	Wild type Cas1-Cas2 expression plasmid under control of the araBAD promoter. *E. coli ygbT* and *ygbF* cloned into pBadHisA [5].
pC-termBioID2	pTK91	8 × GGS linker BioID2 expression plasmid. *Aquifex aeolicus birA* R40G codon optimized for *E. coli* synthesized by GeneArt™ (ThermoFisher Scientific™) with and 8 × GGS linker before the start codon and flanked by NcoI and KpnI restriction sites. Inserted into pBadHisA.
pBioID2	pTK92	BioID2 expression plasmid under control of the araBAD promoter. *Aquifex aeolicus birA* R40G codon optimized for *E. coli* synthesized by GeneArt™ (ThermoFisher Scientific™). Inserted into pBadHisA with NcoI and KpnI restriction sites.
pCas1^{BioID2}-Cas2	pTK134	Cas1 fused at the C-terminus with BioID2 via a 8 × GGS linker, alongside Cas2, all under control of the L-arabinose inducible araBAD promoter. PCR fragments were produced by amplifying pEB628 with primers oTK92F/R and pTK91 with primers oTK93 F/R. Fragments were assembled using the NEBuilder® Hi-Fi DNA Assembly kit (New England Biolabs®). Plasmid is a pBadHisA backbone.
pCas1^{Strep}-Cas2	pTK37	Cas1 with a C-terminal Strep-TagII®, alongside Cas2 both under control of the L-arabinose inducible araBAD promoter. pEB628 PCR amplified with primers oTK02 F/R to insert a Strep-TagII® before the stop codon of *E. coli ygbT*.

pCas1R84G-Cas2	pTK145	Cas1R84G that is defective in acquisition and unable to bind DNA [5, 6], alongside Cas2, both under control of the L-arabinose inducible araBAD promoter. pEB628 mutated with primers Arg84 F/R.
pCas1^{eYFP}-Cas2	pTK135	Cas1 fused at the C-terminus with eYFP via a 8 × GGS linker, alongside Cas2, all under control of the L-arabinose inducible araBAD promoter. PCR fragments were produced by amplifying pTK134 with primers oTK111 F/R to remove BioID2, and pLau18 with primers oTK112 F/R to amplify eYFP. Fragments were assembled using the NEBuilder® Hi-Fi DNA Assembly kit (New England Biolabs®). Plasmid is a pBadHisA backbone.
pCas1R84G^{eYFP}-Cas2	pTK136	As described for pCas1^{eYFP}-Cas2, but with acquisition and DNA binding deficient Cas1R84G. pTK135 pTK135 site directed mutagenesis with primers Arg84 F/R
pDnaK	pTK82	Wild type DnaK with an N-terminal 6 × HisTag under control of an IPTG inducible T7 promoter. *E. coli dnaK* amplified from purified MG1655 genomic DNA using oTK38 F/R inserted into pACYCduet between BamHI and HindIII.
pHtpG	pTK147	Wild type HtpG with an N-terminal 6 × HisTag under control of an IPTG inducible T7 promoter. *E. coli htpG* amplified from purified MG1655 cgenomic DNA using oTK131 F/R inserted into pACYCduet with BamHI and HindIII.
pSecB	pTK131	Wild type SecB under control of an IPTG inducible T7 promoter. *E. coli secB* amplified from purified MG1655 genomic DNA using primers oTK103 F/R inserted into pACYCduet with NcoI and HindIII.
pDnaK^{E171A}	pTK129	DnaK E171A ATPase inactive veriant with N-terminal 6 × HisTag under control of an IPTG inducible T7 promoter. pTK82 site directed mutagenesis with primers oTK101 F/R.

pDnaK^{S427P}	pTK144	DnaK S427P deficient in peptide binding[9] with N-terminal 6 × HisTag under control of an IPTG inducible T7 promoter. pTK82 site directed mutagenesis with primers oTK108 F/R.
pDnaK^{N451K}	pFK08	DnaK N451K deficient in peptide binding[9] with N-terminal 6 × HisTag under control of an IPTG inducible T7 promoter. pTK82 site directed mutagenesis with primers oTK153 F/R.
pDnaK + λ P	pTK149	Wild type DnaK with an N-terminal 6 × HisTag under control of an IPTG inducible T7 promoter in MCS1 and λ phage P gene under control of a seperate IPTG inducible T7 promoter. The λ phage P gene was amplified from purified lambda DNA using primers oTK132 F/R and inserted into pTK82 using KpnI and PacI restriction sites.
pDnaK + λ V	pTK150	Wild type DnaK with an N-terminal 6 × HisTag under control of an IPTG inducible T7 promoter in MCS1 and λ phage V gene under control of a seperate IPTG inducible T7 promoter. The λ phage V gene was amplified from purified lambda DNA using primers oTK133 F/R and inserted into pTK82 using KpnI and PacI restriction sites.
pDnaK + DnaB	pTK156	Wild type DnaK with an N-terminal 6 × HisTag under control of an IPTG inducible T7 promoter in MCS1 and *E. coli* DnaB under control of a seperate IPTG inducible T7 promoter. The *dnaB* gene was amplified from purified *E. coli* genomic DNA using primers oTK162 F/R and inserted into pTK82 using NdeI and XhoI restriction sites.
pMR6		Wild type DnaK expression plasmid under control of araBad promoter. *E. coli dnaK* amplified from MG1655 genomic DNA using dnaKpBad F/R primers inserted into pBadHisA using EcoRI restriction site.
pCas1^{Strep}	pTK 59	Cas1 with a C-terminal Strep-TagII® under control of a T7 promoter. *E. coli ygbT* synthesised by GeneArt™ (ThermoFisher Scientific™) with a 3 ′ thrombin site and Strep-Tag II® before the stop codon, flanked by NdeI and

		XhoI restriction sites. Inserted into pETDuet with NdeI and XhoI.
pCas2^{Strep}	pTK60	Cas2 with a C-terminal Strep-TagII® under control of a T7 promoter. *E. coli ygbF* synthesised by GeneArt™ (ThermoFisher Scientific™) with a 3 ′ thrombin site and Strep-Tag II® before the stop codon, flanked by NdeI and XhoI restriction sites. Inserted into pETDuet with NdeI and XhoI.
pCRISPR	pJRW2	*E. coli* CRISPR-1 cloned into pBluescript [5, 6].

Supplementary Table 10: PCR Primers used in this study

PCR Primer	Sequence (5' - 3')
5'BsrGI-ygbT	TGGACGAGCTGTACAAGATGACCTGGCTTCCCCTTAA
3'ygbT-XbaI	CGCATGTCTAGATCAGCTACTCCGATGGCCTG
5'dnaN_F	TAAGATCGAGCAGTTGCGT
3'dnaN_R	AATCGGTAGCGTAGGACGA
Arg84 F	TGGAGGTGCGGGTTCAGATAAG
Arg84 R	GGCTGACCAGAAGCATAAAC
dnaKdelF	CAGACTCACAACCACATGATGACCGAATATATAGTGGAGACGTTTAGAT GATTCCGGGGATCCGTCGACC
dnaKdelR	GTCAGTATAATTACCCGTTTATAGGGCGATTATTTTTTGTCTTTGACTTCT GTAGGCTGGAGCTGCTTCG
dnaKpBadF	CTGCAGCTGGTACCATATGGATATGGGTAAAATAATTGGTATCG
dnaKpBadR	CGCCAAAACAGCCAAGCTTCTTATTTTTTGTCTTTGACTTCTTC
dnaJ-catdel-fw	ATGGCTAAGCAAGATTATTACGAGATTTTAGGCGTTTCCAAAACAGCGGA GTGTAGGCTGGAGCTGCTT
dnaJ-catdel-rv	TTAGCGGGTCAGGTCGTCAAAAAACTTCTTCACACCATCAAAGAAGCTCT CCATATGAATATCCTCCTTAGT
oTK02F	CAGTTTGAAAAATGAAATGAGTATGTTGGTCGTGG
oTK02R	CGGATGGCTCCAGCTACTCCGATGGCCTGC
oTK38 F	GCATGGATCCGGGTAAAATAATTGGTATCGACCTGGGT
oTK38 R	GCATAAGCTTTTATTTTTTGTCTTTGACTTCTTCAAATTCAGCGTC
oTK92 F	CCTGCGTCGTAGCTGAAATGAGTATGTTGGTCGTGG
oTK92 R	CACCGCTACCACCGCTACTCCGATGGCCTGC
oTK93 F	CATCGGAGTAGCGGTGGTAGCGGTGGTTCAG
oTK93 R	ATACTCATTTCAGCTACGACGCAGGCTAAATTC
oTK101 F	ATCATCAACGCACCGACCGCA
oTK101 R	ACGTTTTACTTCCAGACCAG
oTK103 F	GCATCCATGGATGTCAGAACAAAACAACACTGAAATGACTTT
oTK103 R	GCATAAGCTTTCAGGCATCCTGATGTTCTTCAGT
oTK108 F	CCAGGTGTTCCCGACCGCTGAAG
oTK108 R	CTGTGCTTGGTCGGGATA

oTK111 F	GAGCTGTACAAGTGAAATGAGTATGTTGGTCGTGGTCAC
oTK111 R	GCCCTTGCTCACGCTGCCACCACTGCCACC
oTK112 F	CAGTGGTGGCAGCGTGAGCAAGGGCGAGGAG
oTK112 R	CATACTCATTTCACTTGTACAGCTCGTCCATGC
oTK131 F	ATGCGGATCCGAAAGGACAAGAAACTCGTGGTTTTCAG
oTK131 R	ATGCAAGCTTTCAGGAAACCAGCAGCTGGT
oTK132 F	GCATGGTACCAAAAACATCGCCGCACAGATGG
oTK132 R	GCATTTAATTAATCATACACTTGCTCCTTTCAGTCCG
oTK133 F	GCATGGTACCCCTGTACCAAATCCTACAATGCCG
oTK133 R	GCATTTAATTAATTAACTGGCGGTGACGGTAATTTCT
oTK153 F	CGGCTGATAAAAAATCTCTGGG
oTK153 R	CACGTTTACGTTCACCCTG
oTK162 F	CGATCATATGGCAGGAAATAAACCCTTCAACAAAC
oTK162 R	GCATCTCGAGTTATTCGTCGTCGTACTGCGGC
SW1	AGCTGATCTTTAATAATAAGGAAAT
SW2	CGGATTTATAAAGCTGACGGTT

Supplementary Table 11: Oligonucleotides used in this study to generate DNA substrates.

Substrate	Oligo	Sequence (5′ - 3′)
Pre-spacer	TK24	GCAGTCCCCTCGCCTCAGCTACGCTCGT
	TK25	Cy5-CGTAGCTGAGGCGAGGGGACTGCTGGGC
Fork	MW12	GTCGGATCCTCTAGACAGCTCCATGATCACTGGCACTGGTAGAATTCGGC
	MW14	Cy5-<br>CAACGTCATAGACGATTACATTGCTACATGGAGCTGTCTAGAGGATCCGA

Supplementary References

1. Reyes-Lamothe, R., D.J. Sherratt, and M.C. Leake, *Stoichiometry and architecture of active DNA replication machinery in Escherichia coli.* Science, 2010. **328**(5977): p. 498-501.
2. Rudolph, C.J., A.L. Upton, and R.G. Lloyd, *Replication fork stalling and cell cycle arrest in UV-irradiated Escherichia coli.* Genes Dev, 2007. **21**(6): p. 668-81.
3. Datsenko, K.A. and B.L. Wanner, *One-step inactivation of chromosomal genes in Escherichia coli K-12 using PCR products.* Proc Natl Acad Sci U S A, 2000. **97**(12): p. 6640-5.
4. Stockum, A., R.G. Lloyd, and C.J. Rudolph, *On the viability of Escherichia coli cells lacking DNA topoisomerase I.* BMC Microbiol, 2012. **12**: p. 26.
5. Ivancic-Bace, I., et al., *Different genome stability proteins underpin primed and naive adaptation in E. coli CRISPR-Cas immunity.* Nucleic Acids Res, 2015. **43**(22): p. 10821-30.
6. Radovcic, M., et al., *CRISPR-Cas adaptation in Escherichia coli requires RecBCD helicase but not nuclease activity, is independent of homologous recombination, and is antagonized by 5' ssDNA exonucleases.* Nucleic Acids Res, 2018.
7. He, L., et al., *Interaction of human HelQ with DNA polymerase delta halts DNA synthesis and stimulates DNA single-strand annealing.* Nucleic Acids Res, 2023.

Figure S1

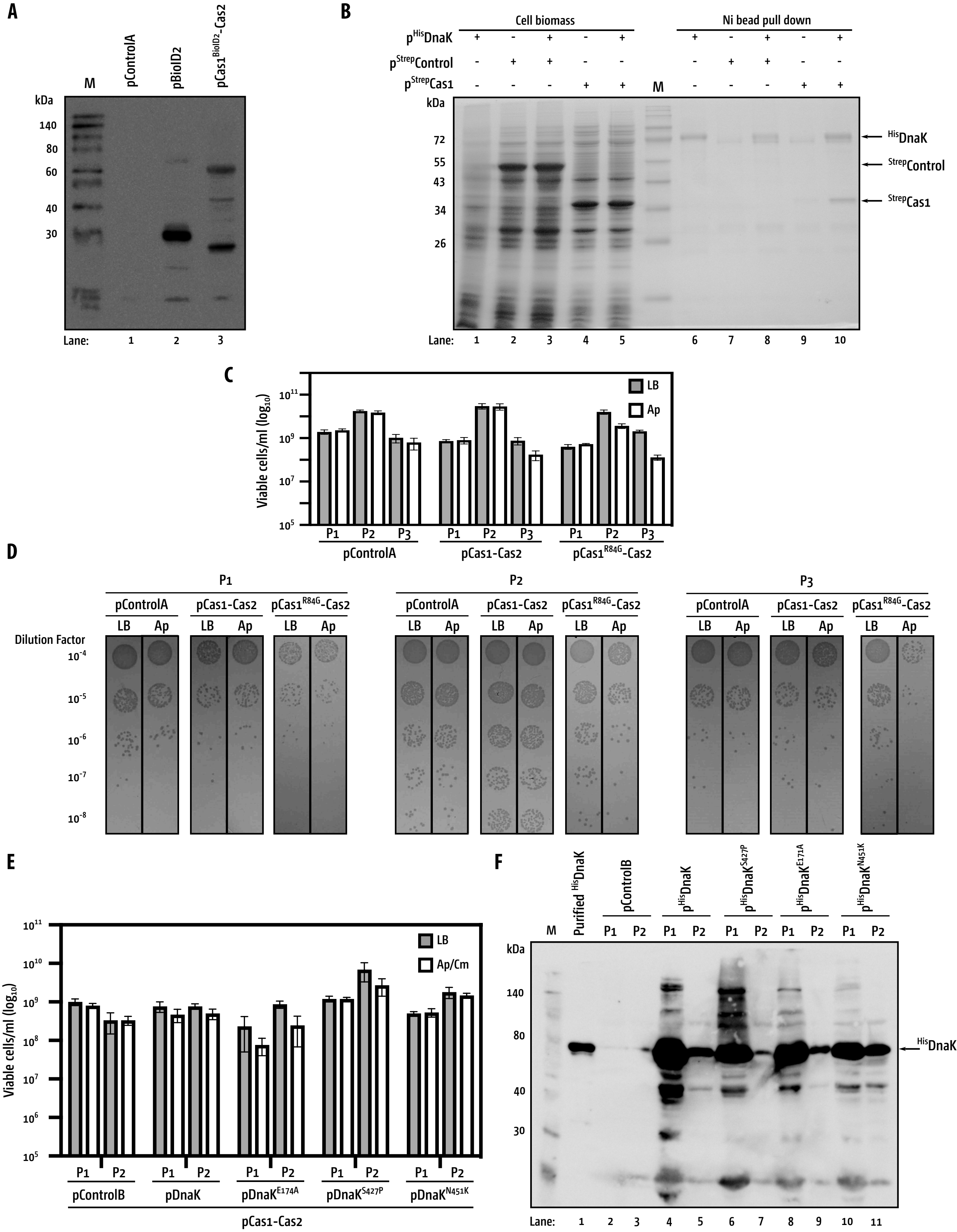

A
M
pControlA
pBioID2
pCas1^{BioID2}-Cas2
kDa
140
80
60
40
30
Lane: 1 2 3
B
Cell biomass
Ni bead pull down
p^{His}DnaK
p^{Strep}Control
p^{Strep}Cas1
M
kDa
72
55
43
34
26
^{His}DnaK
^{Strep}Control
^{Strep}Cas1
Lane: 1 2 3 4 5 6 7 8 9 10
C
Viable cells/ml (log_{10})
LB
Ap
P1 P2 P3
pControlA
pCas1-Cas2
pCas1^{R84G}-Cas2
D
P1
P2
P3
Dilution Factor
10^{-4}
10^{-5}
10^{-6}
10^{-7}
10^{-8}
LB Ap
E
Viable cells/ml (log_{10})
LB
Ap/Cm
pControlB
pDnaK
pDnaK^{E174A}
pDnaK^{S427P}
pDnaK^{N451K}
pCas1-Cas2
F
M
Purified ^{His}DnaK
pControlB
p^{His}DnaK
p^{His}DnaK^{S427P}
p^{His}DnaK^{E171A}
p^{His}DnaK^{N451K}
P1 P2
kDa
140
80
40
30
^{His}DnaK
Lane: 1 2 3 4 5 6 7 8 9 10 11

Figure S2

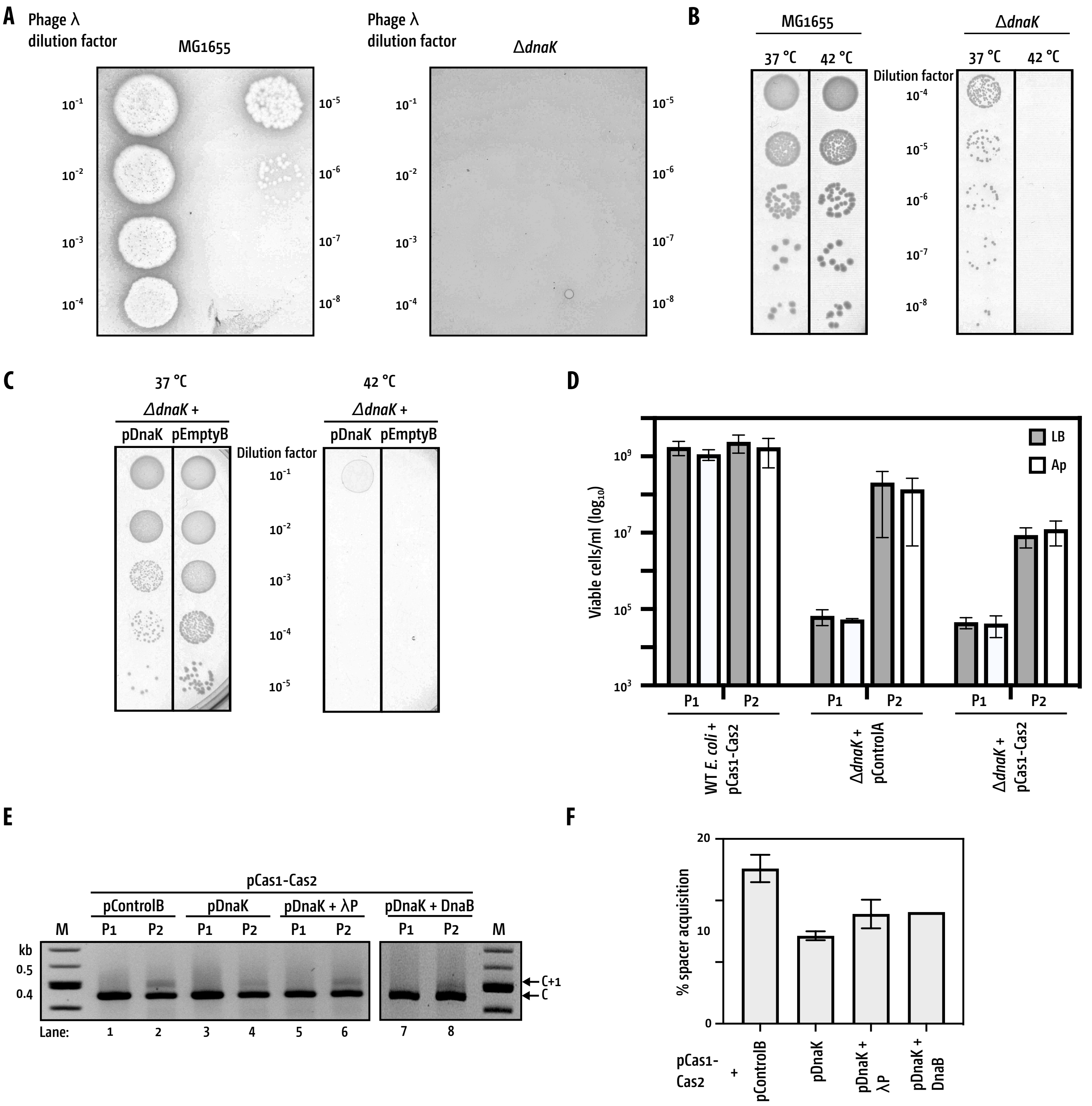

A
Phage λ dilution factor
MG1655
10^{-1}
10^{-2}
10^{-3}
10^{-4}
10^{-5}
10^{-6}
10^{-7}
10^{-8}
Phage λ dilution factor
ΔdnaK
B
MG1655
ΔdnaK
37 °C
42 °C
Dilution factor
C
37 °C
42 °C
ΔdnaK +
pDnaK
pEmptyB
Dilution factor
D
LB
Ap
Viable cells/ml (log_{10})
10^9
10^7
10^5
10^3
P1
P2
WT E. coli + pCas1-Cas2
ΔdnaK + pControlA
ΔdnaK + pCas1-Cas2
E
pCas1-Cas2
pControlB
pDnaK
pDnaK + λP
pDnaK + DnaB
M
kb
0.5
0.4
C+1
C
Lane: 1 2 3 4 5 6 7 8
F
% spacer acquisition
20
10
0
pCas1-Cas2 +
pControlB
pDnaK
pDnaK + λP
pDnaK + DnaB

Figure S3

ΔdnaK
eYFP only

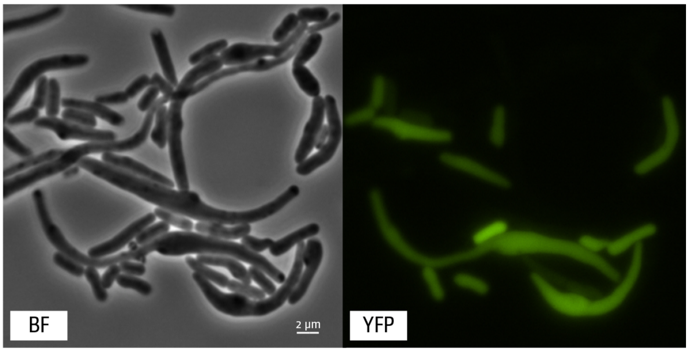

Figure S4

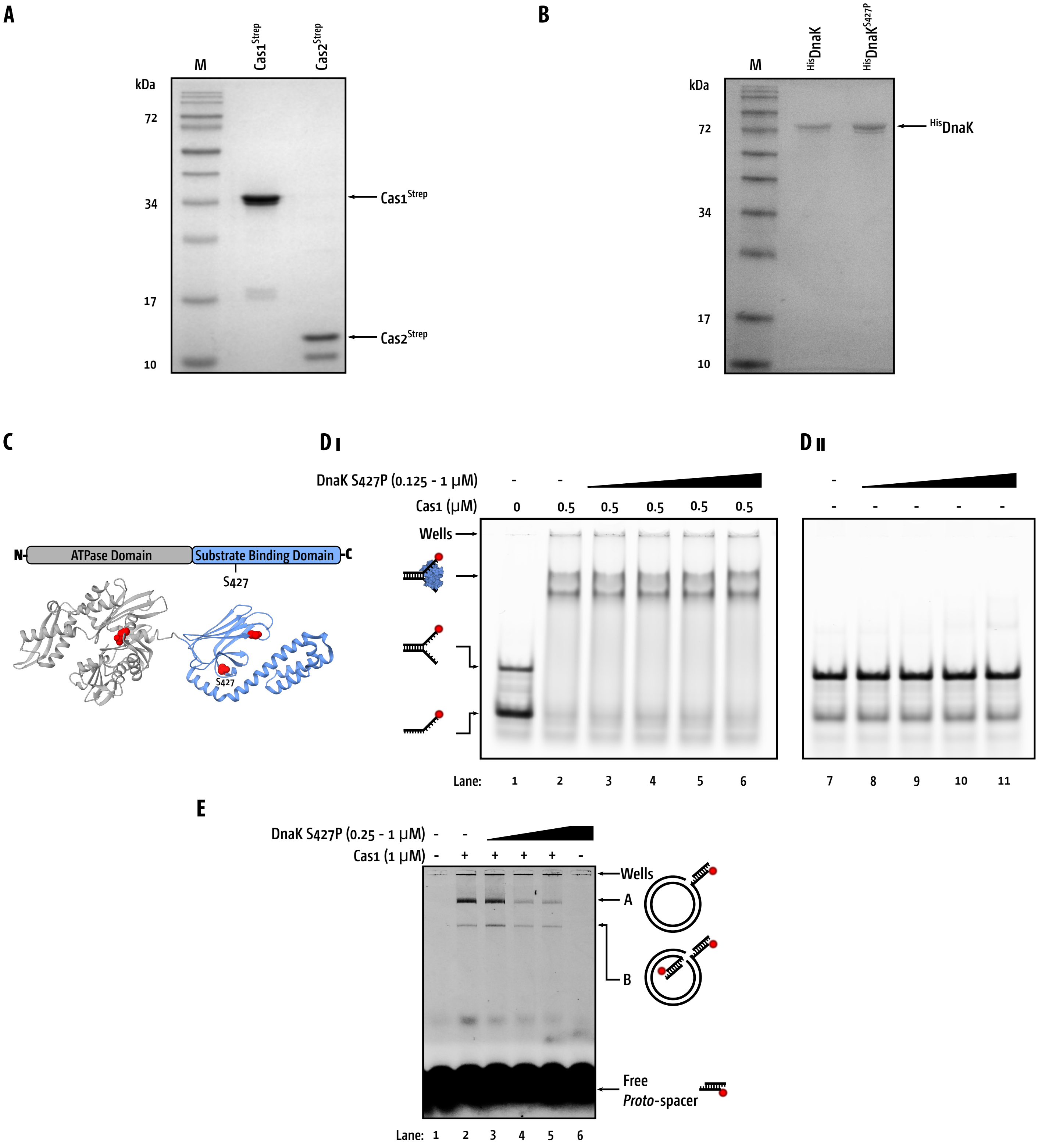

A
M
Cas1Strep
Cas2Strep
kDa
72
34
17
10
Cas1Strep
Cas2Strep
B
M
HisDnaK
HisDnaKS427P
kDa
72
34
17
10
HisDnaK
C
N-
ATPase Domain
Substrate Binding Domain
-C
S427
S427
D I
DnaK S427P (0.125 - 1 μM)
Cas1 (μM)
0
0.5
0.5
0.5
0.5
0.5
Wells
Lane:
1
2
3
4
5
6
D II
7
8
9
10
11
E
DnaK S427P (0.25 - 1 μM)
Cas1 (1 μM)
Wells
A
B
Free
Proto-spacer
Lane:
1
2
3
4
5
6
